# Pain Management in Italian Elite Athletes: Trends in the Use of Non-Steroidal Anti-Inflammatory Drugs (NSAIDs), Glucocorticoids, and Narcotics in Anti-Doping Reports (2013–2023)

**DOI:** 10.3390/ph19020298

**Published:** 2026-02-11

**Authors:** Mario Ruggiero, Stefania Santamaria, Pietro Montesano, Leopoldo Ferrante, Yuri Russo, Filomena Mazzeo

**Affiliations:** 1 Department of Medical, Human Movement and Well-Being Sciences, University of Naples Parthenope, Via Medina 40, 80133 Napoli, Italy; stefania.santamaria@uniparthenope.it (S.S.); pieromontesano@libero.it (P.M.); 2 Department of Economics, Law, Cybersecurity and Sports Sciences, University of Naples Parthenope, 80035 Nola, Italy; leopoldo.ferrante@collaboratore.uniparthenope.it; 3 Department of Public Health and Sport Sciences, University of Exeter, Exeter EX1 2LU, UK; y.russo@exeter.ac.uk; 4 Department of Law, Economics and Human Sciences (DiGiES), Mediterranea University of Reggio Calabria, Via dell’Università, 25, 89124 Reggio Calabria, Italy

**Keywords:** doping, Italian athletes, pain therapy, non-steroidal anti-inflammatory drugs (NSAIDs), glucocorticoids, narcotics, sport, pharmaceutical

## Abstract

**Background**: Analgesics are widely used in competitive sports, but their patterns of use and detection in anti-doping controls vary significantly across drug classes. This study examined a decade of Italian anti-doping reports with three aims: to describe trends involving non-steroidal anti-inflammatory drugs (NSAIDs), glucocorticoids, and narcotics; to characterize the distribution of specific active ingredients; and to analyze the relative contribution of these classes to anti-doping violations, placing the findings within the regulatory framework. **Methods**: Data from national anti-doping reporting systems were analyzed from 2013 to the first half of 2023. Yearly data included tested athletes, athlete declarations of NSAID use, and laboratory analytical findings for prohibited substances (glucocorticoids and narcotics). NSAID prevalence was calculated relative to tested athletes, while glucocorticoid and narcotic findings were assessed as absolute counts and proportions of total violations. Temporal trends were assessed using the Cochran–Armitage test. **Results**: NSAIDs consistently ranked as the most frequently reported medication, with nearly half of the tested athletes reporting their use and no significant linear trend in overall prevalence. However, a significant shift was observed within the NSAID class, with a marked decrease in declarations of COX-2 selective agents over time. Glucocorticoids accounted for a significant portion of prohibited substances, with fluctuating proportions (showing no significant linear trend), betamethasone being the most common active ingredient. Narcotics appeared only sporadically, although the use of non-prohibited opioids such as tramadol and codeine—absent from official reports—remains relevant for understanding analgesic practices. **Conclusions**: Analgesic use in Italian elite sports shows distinct patterns driven by therapeutic needs and anti-doping regulations. NSAIDs remain the primary choice for routine pain management, though the type of NSAID reported has shifted significantly. Glucocorticoids represent a notable share of prohibited findings with a fluctuating, rather than steadily increasing, pattern. Narcotics appear only sporadically in violation data. Ongoing monitoring will be crucial to understanding how evolving clinical practices and recent regulatory changes influence future detection trends and athlete health.

## 1. Introduction

Pain is a prevalent condition in the general population and is clinically classified into acute pain, typically related to trauma, overload, or transient conditions, and chronic pain, defined as lasting longer than three months. Epidemiological studies show that approximately one-third of adults report a recent episode of pain (episodic/acute pain) within the past week [1] and that about one in three adults experiences recurrent pain over the course of a year [2].

Athletes commonly experience pain as an inherent component of training and competition, with both acute and overuse-related symptoms frequently reported across sport disciplines [3]. Elite athletes operate under complex physiological conditions in which training load, energy balance, endocrine regulation, and recovery dynamics interact closely, with exercise-induced adaptations influencing metabolic, neuroendocrine, and motor control systems [4,5,6]. Epidemiological studies indicate that musculoskeletal pain in athletes is highly prevalent, with point estimates ranging from 30% to over 60% depending on the sport, training load, and assessment method [7]. Pain episodes often arise from acute injuries, repetitive microtrauma, or high mechanical and metabolic demands, and may recur or persist throughout the competitive season. Moreover, athletes frequently train and compete despite experiencing pain, which can contribute to symptom chronicity and may increase reliance on pharmacological strategies for symptom control and performance continuity [8]. External pressures also influence pain management in elite sport. Athletes may feel encouraged to return to play rapidly or to rely on analgesic medications to continue training and competing despite injuries [9,10,11,12]. These dynamics can create situations in which performance expectations and health priorities are difficult to align, increasing the athlete’s vulnerability [13,14,15]. In addition, the dual obligations of team physicians toward both the athlete and the club may generate conflicting expectations, further complicating decisions about pain management [16].

In the general population, first-line pharmacological treatments include Non-Steroidal Anti-Inflammatory Drugs (NSAIDs) for nociceptive pain, with glucocorticoids typically reserved for specific inflammatory conditions or intra-articular injections rather than systemic chronic use [17], antidepressants and antiepileptics for neuropathic pain [18], and opioids for moderate to severe cases of both types, although with risks of tolerance, dependence, and a broad spectrum of adverse effects [19,20].

In contrast, for elite athletes, this pharmacological landscape is fundamentally complicated by the imperative to comply with anti-doping regulations. The increasing use of supplements and ergogenic aids further complicates this landscape, as athletes may be inadvertently exposed to prohibited substances through contaminated products or mislabeled ingredients, highlighting the importance of vigilance in nutritional and supplementation practices [21,22,23,24]. The World Anti-Doping Agency (WADA) Prohibited List severely restricts the use of several key analgesic classes [25,26]. While NSAIDs are permitted due to their peripheral action and favorable risk–benefit profile in this context, the therapeutic arsenal is significantly limited by the prohibition of other key classes: glucocorticoids and narcotics.

NSAIDs are a key component of sports medicine due to their analgesic and anti-inflammatory properties [27]. Their use among athletes is widespread, with daily use in elite athletes reaching 25–35%, far exceeding the 1–4% prevalence in the general population [28]. They exert their effects by inhibiting cyclooxygenase (COX) enzymes and, in some cases, 5-lipoxygenase (5-LOX), which are crucial in arachidonic acid metabolism [29,30]. COX-1 provides constitutive protective functions [31,32], while COX-2 is induced during inflammation [33,34]. Agents vary from non-selective to COX-2 selective (coxibs) [35,36]. Pharmacokinetically, they are rapidly absorbed, highly protein-bound, and metabolized hepatically via cytochrome P450 enzymes (e.g., CYP3A, CYP2C9), leading to variable half-lives and individual responses [37,38,39,40,41]. Despite being permitted, they pose risks of gastrointestinal, renal, and cardiovascular side effects [42,43] and may impair healing in specific musculoskeletal injuries [44,45,46,47]. Therefore, their use in athletes requires clinical guidance and should be integrated into a comprehensive rehabilitation plan [28].

Glucocorticoids are frequently prescribed in sports medicine for musculoskeletal inflammation [35,36,37]. Their mechanism involves genomic regulation: upon binding intracellular receptors, the complex modulates gene transcription, suppressing pro-inflammatory mediators (e.g., COX-2, cytokines) and activating anti-inflammatory proteins [48,49,50]. A key action is the inhibition of phospholipase A2, reducing prostaglandin and leukotriene production [51]. Effects persist beyond plasma detection due to this genomic mode of action [52]. Structurally derived from cholesterol, synthetic modifications enhance receptor affinity and stability [53]. Systemically, they are classified by potency, mineralocorticoid activity, and HPA-axis suppression duration; some require hepatic activation [54]. The anti-inflammatory onset is delayed but potent, and HPA-axis suppression often outlasts the therapeutic effect, complicating dosing [52]. However, their use in competition is prohibited by WADA due to their documented potential to enhance performance. Suspected since the 1960s [55], glucocorticoids may boost performance by increasing the availability of metabolic substrates, such as glucose [56,57], and by exerting central effects that decrease the perception of pain and fatigue, thereby raising the fatigue threshold [58]. Their immunosuppressive and anti-inflammatory properties can also help recovery from exercise-induced muscle damage [59,60]. Their use carries significant risks, including tendon rupture and cartilage damage [61,62]. Because of the high risk of adverse effects, such as muscle wasting, and their potential to improve performance, WADA has established specific washout periods and urinary reporting levels to regulate their use [63].

Narcotics are occasionally used in sports medicine for severe acute pain (e.g., fractures, postoperative). Their analgesia is mediated via the endogenous opioid system, acting on widely distributed G protein–coupled receptors (MOR, KOR, DOR, NOP) in the central and peripheral nervous systems [64,65]. Receptor activation inhibits neuronal excitability, reducing pain transmission [66]. Their broad distribution explains both high analgesic efficacy and diverse systemic effects [67]. Agonists are effective via various routes, with profiles shaped by compound-specific receptor affinity and pharmacokinetics [68,69]. Despite efficacy, use is limited by adverse effects including sedation, respiratory depression, gastrointestinal dysmotility, and risks of tolerance and dependence [70,71]. These effects are a direct consequence of opioid receptor activation in multiple neural and peripheral systems. In elite sport, reported use is very low—less than 1%—a pattern likely influenced by both the potential for harmful side effects and the fact that many opioid medications are prohibited in-competition by the WADA [72]. Furthermore, evidence regarding their efficacy in athletes is limited, and available data suggest that opioid-containing analgesics may provide no functional advantage over NSAIDs while being associated with a higher incidence of adverse events [73].

For an athlete to use prohibited medication for therapeutic reasons, they must obtain a Therapeutic Use Exemption (TUE), which requires demonstrating a proven medical need, a lack of therapeutically equivalent non-prohibited alternatives, no performance advantage, and use of the minimum effective dose [74]. This creates a critical challenge for sports medicine physicians, who must navigate the fine line between ensuring effective pain management to maintain training and competition schedules and adhering strictly to anti-doping rules to avoid therapeutic misuse and potential sanctions.

Building on these considerations, the present study examines the patterns of use of NSAIDs, glucocorticoids, and narcotics among Italian elite athletes by analyzing anti-doping reports issued by the Italian Ministry of Health over a decade, from 2013 to the first half of 2023 (FH2023) [75]. This descriptive epidemiological analysis aims to: (1) Describe the annual prevalence and temporal trends in NSAID declarations and in the detection of glucocorticoids and narcotics (as adverse analytical findings) among tested athletes; (2) characterize the distribution of specific active ingredients reported or detected within each of the three pharmacological classes; (3) analyze the relative contribution of glucocorticoid and narcotic findings to the total number of anti-doping rule violations over the study period. The goal is to gain insight into how athletes manage pain while operating within the constraints imposed by anti-doping regulations.

## 2. Results

The anti-doping data examined in this study were obtained from the official annual reports published within the Italian Ministry of Health’s “Reporting System Doping Antidoping Archives” [75]. The information extracted covers the period from 2013 to FH2023 and includes the total number of athletes tested each year, the NSAIDs declared at the time of testing, the prohibited substances identified, and, in particular, glucocorticoids and narcotics during laboratory analysis.

During the study, a total of 9882 athletes underwent doping tests, with an annual average of 898.36. Among these athletes, a total of 4292 reported the use of NSAIDs, corresponding to an annual average percentage of 45.34%. Anti-doping analyses also identified a total of 430 prohibited substances throughout the study period, with an annual rate of 39.09 cases. Within this group, glucocorticoid-related findings amounted to a total of 51 cases, corresponding to an annual average percentage of 0.50%, while narcotic-related findings were limited to a total of 3 cases, with an annual average percentage of 0.03% per year.

Table 1 summarizes all values extracted from the anti-doping reports for each year.

**Figure 1:**
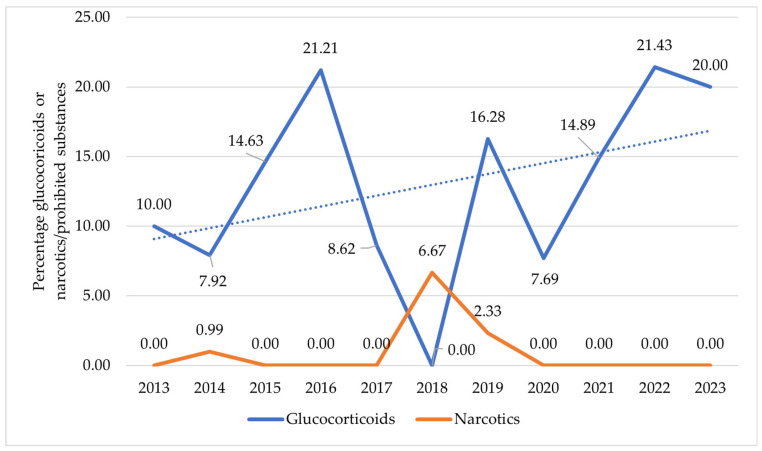
Trend in glucocorticoid and narcotic use among athletes in Italy from 2013 to FH2023. The data, expressed as a percentage, show the proportion of cases related to each substance class relative to the total number of positive findings for all prohibited substances recorded annually/over each period. The dashed blue line shows the trend line for glucocorticoid use over the studied period.

Figure 1 shows the proportion of glucocorticoid- and narcotic-related findings in relation to the total number of prohibited substances detected in anti-doping tests from 2013 to FH2023. The trendline is a simple linear regression line presented for visual illustration.

Glucocorticoids made up an average of 12.97% of all prohibited substances identified. The trendline suggests a gradual upward tendency; however, this trend is based on low absolute numbers and shows considerable yearly fluctuation (e.g., no detections in 2018). In contrast, narcotic-related findings accounted for an average of 0.91% of all prohibited substances. Due to the very limited number of cases recorded over the decade, the pattern observed does not allow for a reliable interpretation of temporal trends.

Table 2 presents the distribution of NSAID active ingredients reported by athletes from 2013 to FH2023, organized according to the same classification used in the anti-doping reports: non-selective COX-1 and COX-2 inhibitors, selective COX-2 inhibitors, and highly selective COX-2 inhibitors. Within the group of non-selective agents, ketoprofen and ibuprofen consistently appear among the most frequently reported substances, followed by diclofenac and nimesulide. The “others” category within non-selective inhibitors shows notably high values across several years; this suggests that additional commonly used NSAIDs—likely including acetylsalicylic acid and naproxen, which are not individually listed in the reports—contribute substantially to this total.

Among selective COX-2 inhibitors, celecoxib and etoricoxib show lower but stable frequencies over the examined years. Overall, the table highlights a marked predominance of non-selective NSAIDs, reflecting their widespread availability and routine use in sports medicine compared with selective agents.

Table 3 summarizes all glucocorticoid findings identified in anti-doping tests from 2013 to FH2023. The substances are presented according to their specific active ingredient, as reported in the official documentation. Betamethasone is the glucocorticoid most frequently detected across the study period, followed by prednisolone and prednisone, which appear with lower but recurrent frequencies. Sporadic findings of methylprednisolone and triamcinolone (including triamcinolone acetonide) are also reported.

Table 4 reports the narcotic-related findings recorded between 2013 and FH2023. Only two active ingredients—methadone and oxycodone—were identified in anti-doping analyses, both appearing with very low frequencies, with several years showing no narcotic findings at all. Notably, substances such as codeine and tramadol do not appear in the table, as they are not included in the WADA Prohibited List and therefore are not classified as doping violations. The limited and irregular distribution observed in the table reflects the minimal involvement of narcotics in anti-doping cases during the study period.

### Statistical Trend Analysis

Temporal trends were assessed using the Cochran–Armitage test for trend. There was no evidence of a linear trend over time in the overall proportion of tested athletes who self-reported any NSAID use (χ^2^ (1) = 0.207, *p* = 0.649). When NSAID declarations were examined by subclass, different patterns emerged. Among all tested athletes, the proportion reporting COX-2 selective NSAIDs (including selective and highly selective agents; Table 2) showed a significant decreasing linear trend over the study period (χ^2^ (1) = 10.083, *p* = 0.002). In contrast, there was no evidence of a linear trend in reports of non-selective NSAIDs (χ^2^ (1) = 1.881, *p* = 0.170). Among athletes who reported any NSAID use, the composition of declared NSAID type also changed over time: the share reporting a COX-2 selective NSAIDs decreased significantly (χ^2^ (1) = 10.665, *p* = 0.001), indicating an increasing predominance of non-selective agents among NSAID users (noting that these categories are complementary in this subset). Finally, there was no evidence of a linear temporal trend in the proportion of tested athletes with an adverse analytical finding for glucocorticoids (χ^2^ (1) = 0.008, *p* = 0.927).

## 3. Discussion

The findings of this study outline a clear distinction between the three pharmacological classes, underpinned by a fundamental methodological difference: NSAID data reflect declared use, while glucocorticoid and narcotic data reflect detected violations. NSAIDs represent by far the most frequently reported medications among athletes undergoing anti-doping testing, maintaining consistently high values throughout the entire decade. Glucocorticoids, although markedly less common in absolute terms, account for a notable proportion of the prohibited substances detected and show a pattern of fluctuation with a suggested gradual increase over time when considered relative to the total number of violations. These trends, however, are based on low absolute numbers and should be interpreted with caution. Narcotics, on the other hand, appear only sporadically, with very few cases identified over the ten years examined. Together, these patterns provide an overview of how different analgesic strategies intersect with anti-doping controls in Italian elite sport, highlighting substantial differences in prevalence, regulatory impact, and potential clinical use across the three classes. Statistical trend analysis further informed these patterns, revealing a significant shift within the NSAID class and confirming the absence of a clear linear trend in glucocorticoid detections.

NSAIDs are the most frequently reported drug class in anti-doping reports, and in the dataset, almost one in two athletes reported using them. This level of use is consistent with observations published in the literature: surveys of elite athletes and at international events report high rates of analgesic use, sometimes exceeding 50% in individual contexts, and meta-analyses/syntheses indicate a point prevalence of around 40–50% in groups of young or junior athletes [72,76,77,78]. While the overall rate of NSAID declarations remained stable without a significant linear trend (*p*-value = 0.649), the composition of reported agents changed markedly over the decade.

The widespread use of NSAIDs may be explained by several factors, including their over-the-counter availability, the perceived safety among athletes and medical staff, and their rapid analgesic effects. In addition, NSAIDs are permitted under anti-doping regulations because current evidence indicates that they do not enhance athletic performance [79,80]. This regulatory context supports not only therapeutic use but also routine or even prophylactic administration before training sessions and competitions.

Although NSAIDs are effective in the short term, frequent or prolonged use carries well-documented risks for athletes: gastrointestinal complications (ulcers, bleeding), acute kidney damage or worsening kidney function in situations of dehydration or prolonged exertion, cardiovascular risks associated with certain active ingredients, and possible negative effects on tissue repair and muscle recovery in certain conditions [81,82,83,84,85].

The underlying mechanisms of these toxicities are multifactorial, primarily resulting from the non-selective inhibition of COX enzymes and the subsequent depletion of protective prostaglandins (PGs) [31]. Gastrointestinal toxicity occurs when COX-1-derived PGs vital for mucosal integrity are inhibited [86,87], a process involving mitochondrial dysfunction and inflammatory cytokine release [88,89]. Renal toxicity stems from the inhibition of vasodilatory PGs (PGE2, PGI2) essential for renal blood flow and glomerular filtration, raising the risk of acute kidney injury during dehydration [90,91,92]. Cardiovascular risks, particularly with COX-2 selective inhibitors, are linked to an imbalance between prostacyclin (PGI2) and thromboxane A2 (TxA2), promoting platelet aggregation and atherothrombotic events [93], and can worsen hypertension [94]. Specific studies in athletic populations report adverse events and warn against routine use without clinical indication [95,96].

Taken together, the widespread use of NSAIDs underscores their central role in routine pain management among elite athletes. Formal trend analysis of active ingredients confirmed a significant decrease in the proportion of athletes declaring COX-2 selective NSAIDs (*p*-value = 0.0015), with a corresponding relative increase in non-selective agents. This shift was consistent both among all tested athletes and within the subset of NSAID declarers (*p*-value = 0.0011), suggesting a change in prescribing or self-selection patterns. However, when shifting from permitted medications to substances classified as prohibited in competition, a markedly different pattern emerges. Glucocorticoids and, to an even greater extent, narcotics occupy a complex position at the intersection between therapeutic need and anti-doping regulation, reflecting both their pharmacological impact and their potential to influence performance.

Glucocorticoid-related findings show a distinct temporal pattern when compared with NSAIDs. As illustrated in Figure 1, their proportion relative to the total number of prohibited substances displays notable fluctuations across the decade, including a marked drop in 2018, when no glucocorticoid violations were reported. A substantial reduction is also evident in 2020, a year in which overall anti-doping activity decreased considerably due to the COVID-19 pandemic, affecting both testing volume and the detection of prohibited substances [97]. Despite these fluctuations, the overall trend suggests a gradual increase in the relative contribution of glucocorticoids over time. However, formal trend analysis found no evidence of a significant linear increase in the proportion of athletes with glucocorticoid findings over the study period (*p*-value = 0.927), supporting the interpretation of a fluctuating rather than a steadily rising pattern. Analysis of active ingredients indicates that betamethasone is by far the most frequently identified compound, followed—at a considerable distance—by prednisolone, prednisone, and occasional detections of methylprednisolone and triamcinolone derivatives. This distribution highlights the prominent role of a limited number of systemic glucocorticoids in anti-doping findings. It is important to note that the proportional trends discussed here are derived from a low number of annual cases (see Table 1) and are therefore inherently unstable and sensitive to small changes in detection rates from year to year.

The regulatory framework has undergone some changes in recent years. As of 1 January 2022, WADA has banned all injectable routes of administration of glucocorticoids during competitions, including intra-articular, peri-articular, epidural, and other local injections, in addition to the oral and rectal routes already banned [98]. These regulatory changes are complemented by washout period guidelines for athletes intending to use glucocorticoids out-of-competition, to avoid detection in subsequent in-competition testing. Importantly, the most recent version of the prohibited list of 2026 specifies that the use of sustained-release glucocorticoid formulations may result in detectable systemic levels beyond the nominal washout period, due to prolonged absorption [99].

Given that our dataset extends only to the first half of 2023, these regulatory changes have had limited time to produce measurable effects in our analysis. Therefore, the trends reported here primarily reflect the pre-2022 regulatory environment. This recent, comprehensive ban represents a significant shift in the anti-doping framework aimed at closing a potential loophole for performance enhancement. Future analyses of anti-doping data beyond 2023 will be decisive to determine whether this policy change leads to a measurable decrease in systemic glucocorticoid detection, a shift towards permitted routes of administration, or unforeseen consequences for athlete health and pain management strategies. Ongoing monitoring is essential to evaluate the real-world impact of this important regulatory evolution. Finally, it should be noted that glucocorticoid formulations administered via inhalation, topical application, eye drops, or otologic routes remain permissible; such uses are not reflected in anti-doping reports and thus would not contribute to our documented findings.

Clinically, corticosteroid therapy carries significant risks proportional to dose and duration [100]. Short-term use can cause hyperglycemia, hypertension, infection risk, and gastrointestinal bleeding [101], while long-term use leads to predictable toxicities such as hypothalamic–pituitary–adrenal (HPA) axis suppression, osteoporosis, immunosuppression, muscle wasting, and metabolic disturbances [52]. Local injections carry risks of infection, soft-tissue atrophy, and accelerated joint degeneration [102,103,104]. For athletes, these risks are particularly consequential: immunosuppression increases infection susceptibility, and musculoskeletal complications like tendon rupture and osteoporosis can directly compromise performance and career longevity [61,62].

Furthermore, systemic corticosteroids exhibit numerous pharmacodynamic and pharmacokinetic drug interactions, many mediated through cytochrome P450 3A4 (CYP3A4) metabolism [105]. Corticosteroids are substrates of CYP3A4, so concomitant use of inhibitors (e.g., ketoconazole, itraconazole, ritonavir, cobicistat, grapefruit juice) can increase corticosteroid exposure and toxicity, whereas inducers (e.g., phenobarbital, rifampin, carbamazepine) can reduce efficacy [106]. Pharmacodynamic interactions include antagonism of antihypertensive and hypoglycemic agents, increased risk of hypokalemia with amphotericin B or diuretics, elevated risk of gastrointestinal bleeding with NSAIDs, and variable effects on warfarin and vaccine response [52]. These interactions could complicate the management of comorbidities or concomitant therapies in athletes, necessitating careful medication review and monitoring. Due to these extensive risks, athletes on corticosteroid therapy, especially systemic or repeated forms, require regular monitoring of metabolic profiles, bone mineral density, and overall physiological impact to balance therapeutic benefits against potential long-term harm.

In contrast with the fluctuating and clinically complex profile observed for glucocorticoids, narcotic-related findings in our dataset remain extremely infrequent, with only isolated cases reported across the entire decade (Table 4). This scarcity appears consistent with international evidence, where the prevalence of opioid use among athletes generally ranges between 3% and 5%, depending on sport discipline, level of competition, and data collection methods [107,108]. These rates are substantially lower than those observed for NSAIDs and glucocorticoids in comparable cohorts, suggesting that prohibited narcotics play only a marginal role in pain management among elite athletes. National differences in opioid-prescribing patterns may contribute to variability between countries, particularly in settings such as the United States or parts of Europe, where opioid consumption in the general population is significantly higher [108,109,110].

A key factor in explaining the near absence of narcotic findings is the regulatory framework. Our dataset includes only substances banned in-competition (e.g., methadone, oxycodone) and excludes allowed opioids like tramadol and codeine [111]. It is important to note that these permitted opioids are reported in the literature to be used in sports to manage pain and fatigue [111,112]. Experimental studies on tramadol have shown mixed results regarding its effects on performance [113,114,115], and both substances pose risks of adverse effects and potential dependency [111,116]. Therefore, the very low rate of narcotic violations observed here reflects the specific list of banned substances and should not be seen as an absence of opioid analgesic use in this athlete population. Therefore, the very low rate of narcotic violations observed here primarily reflects the specific list of banned substances. It should not be interpreted as an absence of opioid analgesic use in this athlete population, as a shift towards permitted alternatives is plausible. This has direct implications for athlete health monitoring, as the use of permitted opioids, while within the rules, still carries significant risks (e.g., dependency, cognitive side effects) that require careful clinical oversight and may not be captured by routine anti-doping surveillance.

The use of opioids, including permitted ones, is not without significant risk. Beyond the potential for addiction involving epigenetic modifications in brain reward circuits [117,118,119], opioids can induce acute and chronic adverse effects mediated by their widespread receptor distribution [67]. Key risks include respiratory depression from brainstem MOR activation [120] and neurological effects such as dizziness, impaired concentration, and delayed reaction time that reflect cortical and subcortical inhibition [121,122,123,124]. In the gastrointestinal tract, opioids cause constipation via enteric nervous system effects [125,126]. These side effects are detrimental to health and can directly impair athletic performance and safety [121].

Furthermore, pharmacokinetic interactions represent an additional layer of risk, particularly for opioids metabolized via the cytochrome P450 system, such as tramadol and codeine. These substances are primarily metabolized by the CYP2D6 enzyme to their active metabolites (O-desmethyltramadol and morphine, respectively) [127]. Genetic polymorphisms in CYP2D6 can lead to poor, intermediate, extensive, or ultrarapid metabolizer phenotypes, causing unpredictable variations in drug efficacy and toxicity. In ultrarapid metabolizers, increased formation of active metabolites may enhance central opioid exposure and toxicity, whereas poor metabolizers may experience reduced analgesic efficacy despite standard dosing [128,129].

While severe events directly attributable to tramadol or codeine are rarely documented in elite sport, their potential for addiction, cognitive impairment, and unsafe compensatory overexertion highlights the need for careful clinical oversight and continuous monitoring of their use in high-performance settings. This discussion must also be considered within the broader public health context of the opioid crisis, particularly in the United States, where prescription opioid misuse and overdose deaths have risen dramatically over the past two decades [130].

Although current evidence does not indicate that elite athletes are experiencing opioid-related morbidity or mortality at levels comparable to the general population, isolated severe cases demonstrate the potential for harm. The opioid-related death of MLB pitcher Tyler Skaggs is the most widely cited example, underscoring how opioid misuse—even when occurring outside the formal medical environment—can have catastrophic outcomes [131]. For athletes, the acute adverse effects of opioids represent not only a health risk but also a direct threat to safety.

In summary, the patterns observed across NSAIDs, glucocorticoids, and narcotics highlight distinct roles and regulatory implications for each class within elite sport. These differences reflect not only pharmacological characteristics and therapeutic needs but also the evolving anti-doping landscape.

### Limitations and Future Perspectives

A key factor in interpreting the results is the inherent difference between the data sources. The high frequency of NSAIDs is due to athlete declarations, which record all reported use of this permitted medication class. In contrast, the figures for glucocorticoids and narcotics rely only on adverse analytical findings, which reflect instances of use that broke in-competition rules. While these metrics are not directly comparable as measures of overall use, they are precisely the metrics relevant to the anti-doping framework we are analyzing. One shows the extent of routine, permitted analgesic use (declarations), while the other tracks rule violations (adverse findings). This distinction is central and accurately reflects the different regulatory status of these drug classes, but it prevents a single assessment of total analgesic use prevalence from this dataset.

Another limitation relates to how we interpret trends involving glucocorticoids and narcotics. Anti-doping data only show substances detected during official tests and do not include therapeutic use outside the in-competition period or the use of non-prohibited opioids like tramadol and codeine. Consequently, the available data offer an incomplete picture of the wider analgesic landscape in elite sports.

Moreover, the study includes data only up to FH2023; as a result, the effects of the regulatory changes introduced by WADA in 2022, especially the ban on all injectable glucocorticoid routes during competition, cannot yet be accurately evaluated. Future analyses will benefit from more recent reports, which may shed light on how these regulatory changes impact detection patterns, therapeutic practices, and potential shifts in the prevalence of systemic glucocorticoid use. Ongoing monitoring will also be crucial to understand whether the recent focus on washout periods and the revised guidance on sustained-release formulations influence the frequency or nature of adverse analytical findings.

Finally, expanding surveillance to include a broader range of therapeutic agents, improving the classification of NSAIDs within official reports, and integrating clinical or prescription data where possible would enhance the capacity to interpret analgesic use patterns in high-performance settings. Such developments would contribute to a more comprehensive understanding of how therapeutic needs, health risks, and evolving regulations interact within modern elite sport in Italian contests.

## 4. Materials and Methods

This study constitutes a descriptive epidemiological analysis of administrative anti-doping data. The analysis is based on the Reporting System Doping Antidoping Archives, which compiles the annual anti-doping reports published by the Italian Ministry of Health [75]. The reports include information on athletes tested for doping during events organized by Federazioni Sportive Nazionali (FSN), Discipline Sportive Associate (DSA), and Enti di Promozione Sportiva (EPS). The present analysis considered all available reports from 2013 to the first half of 2023 (FH2023). A central methodological distinction is that data for NSAIDs rely on athlete declarations at the time of testing, reflecting reported use, whereas data for glucocorticoids and narcotics derive exclusively from laboratory detection of prohibited substances (adverse analytical findings).

To ensure clarity, all proportions reported in this study are calculated using explicit, consistent denominators. For prevalence calculations related to athletes, the denominator is always the total number of athletes tested for doping in the corresponding year. This applies to the percentage of athletes declaring NSAID use and the percentage with findings for glucocorticoids or narcotics. For analyzing the composition of anti-doping violations, the denominator is the total number of prohibited substances detected in the corresponding year. This is used to calculate the proportion of all violations attributable to glucocorticoids or narcotics (Figure 1).

The evaluation was conducted in three sequential steps. First, for each year, the number of athletes who declared the use of NSAIDs and the number of glucocorticoid- and narcotic-related violations were expressed as percentages relative to the corresponding annual total of athletes tested. This allowed a direct comparison of the prevalence of each medication class within the population of athletes undergoing anti-doping controls.

Second, findings related to glucocorticoids and narcotics were analyzed in relation to the total number of prohibited substances detected each year. These values were plotted to determine the proportions shown in the graphical analysis, which was also used to explore potential temporal trends by applying a linear trendline.

Third, the active ingredients within each pharmacological class were analyzed. For NSAIDs, substances were grouped into non-selective COX-1 and COX-2 inhibitors, selective COX-2 inhibitors, and highly selective COX-2 inhibitors based on the classification used in the anti-doping reports. All active ingredients detected from 2013 to FH2023 were counted and reported to show their distribution over time. A similar method was applied to glucocorticoids and narcotics, where each specific compound detected as a prohibited substance was recorded and reported annually.

All results shown in tables and figures are based solely on data from official anti-doping reports. As is typical with analyses using administrative records, the study is limited by changes in reporting detail over the years, decreased testing in 2020, and the lack of information on therapeutic indications, dosing schedules, or methods of administration.

### Statistical Analysis

Annual aggregated data were analyzed across the observation period (2013–2022). Because data available for 2023 refer only to the first half of the year (i.e., a partial year), we performed sensitivity analyses and, for the primary analyses, excluded this period, as some conclusions could be driven by incomplete annual ascertainment. For each year, we computed proportions using the relevant yearly denominators and evaluated temporal trends using the Cochran–Armitage test for trend across ordered years. This procedure tests the null hypothesis of no linear (i.e., monotone) change in a binomial proportion over time. Test statistics were reported as a chi-square value (χ^2^) with the corresponding two-sided *p*-value; direction of change (increasing vs. decreasing) was determined based on the observed proportions over time. Trend analyses were conducted for: (1) NSAID self-reporting prevalence among all tested athletes; (2) class-specific prevalence among all tested athletes for non-selective COX inhibitors and for COX-2 selective agents; (3) composition among NSAID declarers, evaluated as the proportion of NSAID declarers reporting a COX-2 selective agent; and (4) glucocorticoid findings among all tested athletes. Statistical significance was assessed at α = 0.05, with exact *p*-values reported.

The trendline shown in Figure 1 was generated using simple linear regression and is presented for visual illustration of the proportional changes over time. Formal statistical inference regarding trends is based on the Cochran–Armitage test results reported in the Statistical Trend Analysis section of the Results.

## 5. Conclusions

This study provides an overview of analgesic use and related anti-doping findings in Italian elite sport over ten years, from 2013 to FH2023, highlighting substantial differences among NSAIDs, glucocorticoids, and narcotics. NSAIDs emerge as the most frequently reported medications, reflecting their accessibility, permissibility, and widespread therapeutic use. Glucocorticoids, although less common in absolute terms, account for a meaningful proportion of adverse analytical findings and exhibit variable temporal patterns influenced by both clinical practice and evolving regulatory frameworks, though these patterns should be interpreted with caution due to the low annual case numbers. Narcotics appear only sporadically in anti-doping results, although the parallel use of non-prohibited opioids such as tramadol and codeine—unreported in official documents—remains an important consideration when interpreting the broader analgesic landscape.

Overall, the findings highlight the complex interaction between therapeutic needs, pharmacological risks, and anti-doping regulations in high-performance sports. They also stress the importance of ongoing monitoring and updated clinical guidelines, especially as recent and upcoming regulatory changes may affect future detection patterns. In this context, the Italian anti-doping system provides a valuable foundation for tracking how evolving rules, medical practices, and athlete behaviors influence analgesic use trends over time.

## Figures and Tables

**Table 1:** Annual number of athletes tested for doping from 2013 to the first half of 2023 (FH2023). NSAID declarations, glucocorticoid- and narcotic-related findings, prohibited substances identified, and positive athletes are reported. NSAID declarations and glucocorticoid and narcotic findings are presented both as absolute numbers and as a percentage of the total number of athletes tested that year.

Year	Athletes Tested for Doping	NSAIDs n (%)	Glucocorticoids n (%)	Narcotics n (%)	Prohibited Substances	Positive Athletes
2013	1390	613 (44.10)	6 (0.43)	0 (0.0)	60	39
2014	1427	573 (40.15)	8 (0.56)	1 (0.07)	101	58
2015	860	349 (40.58)	6 (0.70)	0 (0.0)	41	25
2016	806	390 (48.39)	7 (0.87)	0 (0.0)	33	33
2017	1211	506 (41.78)	5 (0.41)	0 (0.0)	58	58
2018	594	321 (54.04)	0 (0.00)	1 (0.17)	15	15
2019	1245	556 (44.66)	7 (0.56)	1 (0.08)	43	43
2020	395	184 (46.58)	1 (0.25)	0 (0.0)	13	13
2021	1322	481 (36.38)	7 (0.53)	0 (0.0)	47	38
2022	364	172 (47.25)	3 (0.82)	0 (0.0)	14	12
FH2023	268	147 (54.85)	1 (0.37)	0 (0.0)	5	5
2013-FH2023	9882	4292 (43.43)	51 (0.52)	3 (0.03)	430	339

**Table 2:** Distribution of NSAID active ingredients reported by athletes from 2013 to the first half of 2023 (FH2023), classified as non-selective COX-1 and COX-2 inhibitors, selective COX-2 inhibitors, and highly selective COX-2 inhibitors. The table provides a descriptive overview of the specific active ingredients declared.

	Non-Selective COX-1 and COX-2 Inhibitors			Selective COX-2 Inhibitors			Highly Selective COX-2 Inhibitors	
Year	Ibuprofen	Ketoprofen	Others *	Diclofenac	Nimesulide	Others	Celecoxib	Etoricoxib
2013	91	154	239	55	69	4	0	1
2014	75	158	211	65	52	6	2	4
2015	44	117	112	43	26	3	1	3
2016	57	93	150	57	26	3	0	4
2017	93	152	163	63	29	2	1	3
2018	80	96	100	34	8	1	0	2
2019	99	163	210	64	17	1	0	2
2020	40	46	70	22	3	2	0	1
2021	92	106	182	82	13	3	1	2
2022	60	34	55	18	3	1	0	1
FH2023	40	28	57	12	8	0	1	1
2013-FH2023	771	1147	1549	515	254	26	6	24

* The “Others” category for non-selective COX inhibitors includes acetylsalicylic acid, naproxen, and other common NSAIDs not individually listed in the source anti-doping reports.

**Table 3:** Glucocorticoid-related findings detected in anti-doping tests from 2013 to the first half of 2023 (FH2023), reported by active ingredient. The table provides a descriptive overview of the specific active ingredients detected as adverse findings.

Year	Betamethasone	Deflazacort	Methylprednisolone	Prednisolone	Prednisone	Triamcinolone	Triamcinolone Acetonide
2013	5	0	0	0	0	0	1
2014	4	0	0	2	2	0	0
2015	4	0	0	1	1	0	0
2016	3	0	1	1	1	0	1
2017	2	0	0	1	1	0	1
2018	0	0	0	0	0	0	0
2019	3	0	1	1	1	0	1
2020	1	0	0	0	0	0	1
2021	3	0	1	1	1	1	0
2022	0	1	0	1	1	0	0
FH2023	0	0	0	1	0	0	0
2013-FH2023	25	1	3	9	8	1	5

**Table 4:** Narcotic-related findings identified in anti-doping analyses from 2013 to the first half of 2023 (FH2023), reported by active ingredient. The table provides a descriptive overview of the specific active ingredients detected as adverse findings.

Year	Methadone	Oxycodone
2013	0	0
2014	0	1
2015	0	0
2016	0	0
2017	0	0
2018	1	0
2019	1	0
2020	0	0
2021	0	0
2022	0	0
FH2023	0	0
2013-FH2023	2	1

## Data Availability

The data that support the findings of this study are available from the corresponding authors, F.M. and M.R., upon reasonable request. The data are not publicly available due to privacy and ethical restrictions.
